# Using speech recognition technology to investigate the association between timing-related speech features and depression severity

**DOI:** 10.1371/journal.pone.0238726

**Published:** 2020-09-11

**Authors:** Mao Yamamoto, Akihiro Takamiya, Kyosuke Sawada, Michitaka Yoshimura, Momoko Kitazawa, Kuo-ching Liang, Takanori Fujita, Masaru Mimura, Taishiro Kishimoto

**Affiliations:** Department of Neuropsychiatry, Keio University School of Medicine, Tokyo, Japan; Chiba Daigaku, JAPAN

## Abstract

**Background:**

There are no reliable and validated objective biomarkers for the assessment of depression severity. We aimed to investigate the association between depression severity and timing-related speech features using speech recognition technology.

**Method:**

Patients with major depressive disorder (MDD), those with bipolar disorder (BP), and healthy controls (HC) were asked to engage in a non-structured interview with research psychologists. Using automated speech recognition technology, we measured three timing-related speech features: speech rate, pause time, and response time. The severity of depression was assessed using the Hamilton Depression Rating Scale 17-item version (HAMD-17). We conducted the current study to answer the following questions: 1) Are there differences in speech features among MDD, BP, and HC? 2) Do speech features correlate with depression severity? 3) Do changes in speech features correlate with within-subject changes in depression severity?

**Results:**

We collected 1058 data sets from 241 individuals for the study (97 MDD, 68 BP, and 76 HC). There were significant differences in speech features among groups; depressed patients showed slower speech rate, longer pause time, and longer response time than HC. All timing-related speech features showed significant associations with HAMD-17 total scores. Longitudinal changes in speech rate correlated with changes in HAMD-17 total scores.

**Conclusions:**

Depressed individuals showed longer response time, longer pause time, and slower speech rate than healthy individuals, all of which were suggestive of psychomotor retardation. Our study suggests that speech features could be used as objective biomarkers for the assessment of depression severity.

## Introduction

The number of individuals with mental illnesses, such as major depressive disorder (MDD) and bipolar disorder (BP), is increasing around the world [1]. Globally, 241 million people suffered from MDD in 2017, and it was ranked #3 in years lived with disability (YLD) [2], which makes it one of our most pressing current health issues. As there are no objective biomarkers for depressive symptoms, depression severity assessments are based on subjective reports [3]. The gold-standard assessment tools for depressive symptom severity are the Hamilton Depression Rating Scale (HAMD) [4, 5] and Montgomery Asberg Depression Rating Scale (MADRS) [6]. These rating scales require raters to have a large amount of training and experience [3, 7]. Moreover, these rating scales cannot be completely objective, and they may lead to a systematic bias (e.g., an inflation of the baseline scores due to pressures by study sponsors to accelerate patient enrollment) [7]. To overcome these limitations, objective biomarkers that reflect depression severity are warranted. Because typical depressed individuals speak in a low voice, slowly, hesitatingly, and monotonously [8], researchers have tried to quantify these speech features to assess the severity of depression [9, 10]. Many initial studies in the 1970s and 1980s, which used tape recordings and signal processing, reported that depressed individuals showed slower speech rates and longer pause time than healthy individuals [11, 12]. However, acoustic signal processing technology available at the time was not adequate to cross-validate the subjective observations [7]. Since the early 2000s, technology for eliciting and analyzing speech samples has advanced, and been further refined and validated in the literature [7]. Canizzaro et al. found that a reduced speaking rate had a significant correlation with HAMD scores [13]. Also, Mundt et al. found that depressed individuals who responded to antidepressant treatments showed significantly faster speech rates and shorter pause time after the treatments, while non-responders did not show significant changes in speech [7, 14]. Although these previous studies showed the usefulness of timing-related speech features for the assessment of depression severity, most studies collected speech data from a small sample using a structured or semi-structured interview. Such methods may not be directly applicable to daily clinical settings. On the other hand, this study was conducted in real-world clinical settings with non-structured interviews using automated speech recognition technology, which has the potential for more practical clinical use. We hypothesized: 1) patients with depression speak slower, pause longer, and respond slower than healthy people; 2) the severity of depression is negatively correlated with speech rate, and is positively correlated with pause time and response time; 3) As the depression symptoms become less severe in the individual, speech rate becomes faster, and pause time and response time become shorter.

## Methods and materials

### Study participants

The participants were recruited from inpatients and outpatients at Keio University Hospital and seven collaborative research institutes during the period of May 14, 2016 to Feb 31, 2019. Patients who met the criteria for MDD or BP according to the Diagnostic and Statistical Manual of Mental Disorders, Fifth Edition (DSM-5), were included. This research was done as part of a larger project called The Project for Objective Measures Using Computational Psychiatry Technology (PROMPT). The concept and design of PROMPT is reported elsewhere [15]. PROMPT protocols have been registered with the University Hospital Medical Information Network (UMIN) (UMIN ID: UMIN000023764). This study was approved by the ethics committees of Keio University Hospital and other participating facilities, and performed in accordance with the Declaration of Helsinki. Written informed consent was obtained from all participants.

### Data collection

Speech data was collected through 10-minute, non-structured interviews with a research psychiatrist or psychologist. During the interviews, participants talked about non-specific topics such as their everyday lives, disease symptoms, hobbies, etc., and participants were assessed for depressive symptoms using the HAMD 17-item version (HAMD-17) [5]. Interviews were conducted up to 10 times for each participant, and each interview was conducted at least a week apart for both inpatients and outpatients. For outpatients, each outpatient clinic conducted the interview when patients visited the clinic.

Interviews were conducted in a quiet room. For each interview, a participant and an interviewer were seated face to face across a desk. Two array microphones (Classis RM 30 and RM 30 W, made by Beyerdynamic) were placed facing the participant and interviewer, and the distance between the microphones and the interviewer/interviewee was adjusted to be between approximately 40 cm and 70 cm. Each microphone was connected to an audio interface (Roland, model number: UA—55), and its recorded data were converted into a separated wav file format (PC: Mouse Computer, Inc., model number: NG-N-i 3500 PA 3, OS: Windows 10). We analyzed these audio files using Ami Voice (Advanced Media, Inc.), which is a speech recognition software. The speech data from the recordings were segmented into utterance parts based on the time annotations containing speech time and utterance numbers.

We extracted three timing-related speech features from each speech sample: speech rate, pause time, and response time. Speech rate was defined as the number of words per minute, which was calculated by dividing the participant’s total number of words by the participant’s total speech time during a 10-minute period. Pause time was defined as the interval duration between the end of a participant’s speech segment and the beginning of the participant’s next segment. Response time was defined as the mean pause duration between the end of the interviewer’s utterance and the start of an utterance by the participant. Overlapping voice frames were excluded so that back-channel utterances would not confound response time calculations.

### Statistical analyses

We used IBM SPSS Statistics 24 for statistical analyses and excluded data if it was more than 1.5*the interquartile range (IQR) above the third quartile or below the first quartile. Distributions of all variables were inspected using histograms, q-q plots, and Shapiro-Wilk tests. Because response time and pause time were positively skewed, these variables were natural log-transformed to obtain a normal distribution. First, we conducted analyses of covariance (ANCOVA), including speech rate, response time, and pause time as dependent variables, to examine differences among the three study groups (MDD, BP, and HC). Age, sex, and defined daily doses (DDD) [16], which is to account for the potential effect of medications on speech-related measures, were included as covariates. Second, we conducted multiple linear regression analyses to examine the relationships between HAMD-17 scores and each timing-related speech feature with age, sex, and DDD included as covariates. We used the data sets with the highest HAMD-17 scores for each patient with MDD or BP in these analyses. In the current study, we set YMRS > = 8 and HAMD-17 > = 8 as manic and depressive state respectively. We also conducted the same regression analyses after excluding BP patients with YMRS higher than 8 and when patients were divided into MDD and BP respectively (S1 File). As additional analyses, we conducted Spearman correlation analyses to examine the relationship between the item 8 of HAMD-17 (psychomotor retardation) and each timing-related speech feature for patients with MDD or BP. Finally, we conducted multiple linear regression analyses to investigate the association between within-subject change in timing-related speech features and change in HAMD-17 scores (highest HAMD-17 scores—lowest HAMD-17 scores). Interval between interview days varies from participant to participant. Therefore, age, sex, and interval between interview days were included as covariates. Statistical significance was defined by a p-value of <0.05 (two-tailed). Bonferroni correction was used for multiple comparisons correction. Raw p values are reported in the Results section.

## Results

### Clinical characteristics

During the period from May 2016 to February 2019, we collected 1058 speech data sets from 241 participants (479 from 97 patients with MDD, 295 from 68 patients with BP, and 284 from 76 HC). Of these speech data sets, 85 speech samples were not recorded properly due to issues such as inadequate microphone settings and the loss of speech data or rating data. Thus, the remaining 972 speech data sets from 84 patients with MDD, 68 patients with BP, and 71 HC were used in the analyses. Table 1 represents the participants’ demographics. There were significant differences in mean age among groups (F_2,220_ = 14.8, p <0.001). HC were significantly older than patients with MDD (p <0.001) and patients with BP (p = 0.003).

**Table 1:** Demographics.

	MDD (n = 84)	BP (n = 68)	HC (n = 71)
Age, year: mean (s.d.)	50.0 (15.0)	55.1 (17.0)	64.3 (17.3)
Gender, women/men: n	47/37	38/30	38/33
HAMD-17, mean (range)	17.3 (0–38)	13.4 (1–40)	3.3 (0–11)
Antidepressants, %	91.7	30.9	NA
Antipsychotics, %	51.2	75.0	NA
Anxiolytics-Hypnotics, %	66.7	72.1	NA
Mood Stabilizers, %	11.9	63.2	NA

MDD = major depressive disorder; BP = bipolar disorder; HC = healthy controls; HAMD-17 = Hamilton Depression Rating Scale 17-item version; NA = not applicable.

### Group comparisons among MDD, BP, and HC

**Fig 1:**
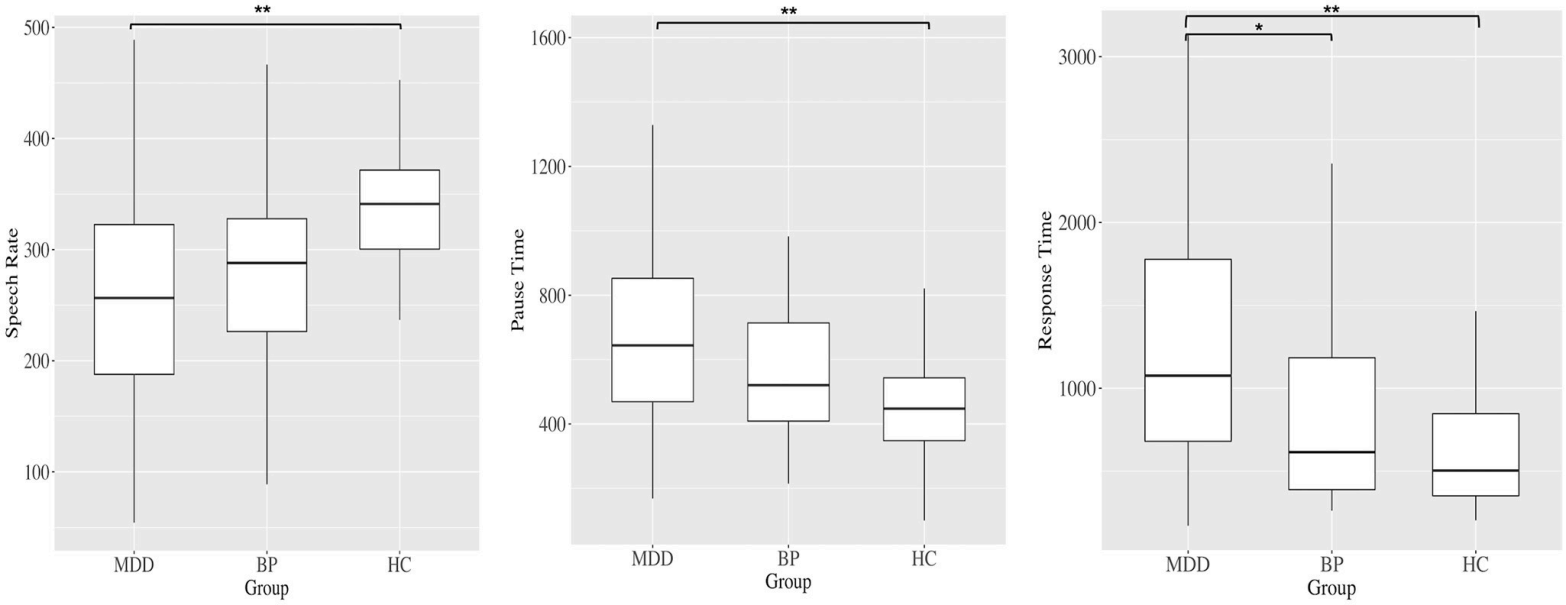
Box plots of speech rate, pause time, and response time. **p<0.01, *p<0.05.

ANCOVA revealed significant differences between study groups in speech rates (F_2,216_ = 6.48, p = 0.002), pause time (F_2,199_ = 5.99, p = 0.003), and response time (F_2,197_ = 9.89, p <0.001) (Fig 1). Patients with MDD had slower speech rates (p = 0.001), longer pause time (p = 0.002), and longer response time (p = 0.001) than HC. Moreover, patients with MDD showed longer response time (p = 0.001) than patients with BP, but there were no significant differences in speech rate (p = 0.498) and pause time (p = 0.314).

There were no significant differences in any speech features between patients with BP and HC.

### Association between HAMD-17 scores and timing-related speech features

**Fig 2:**
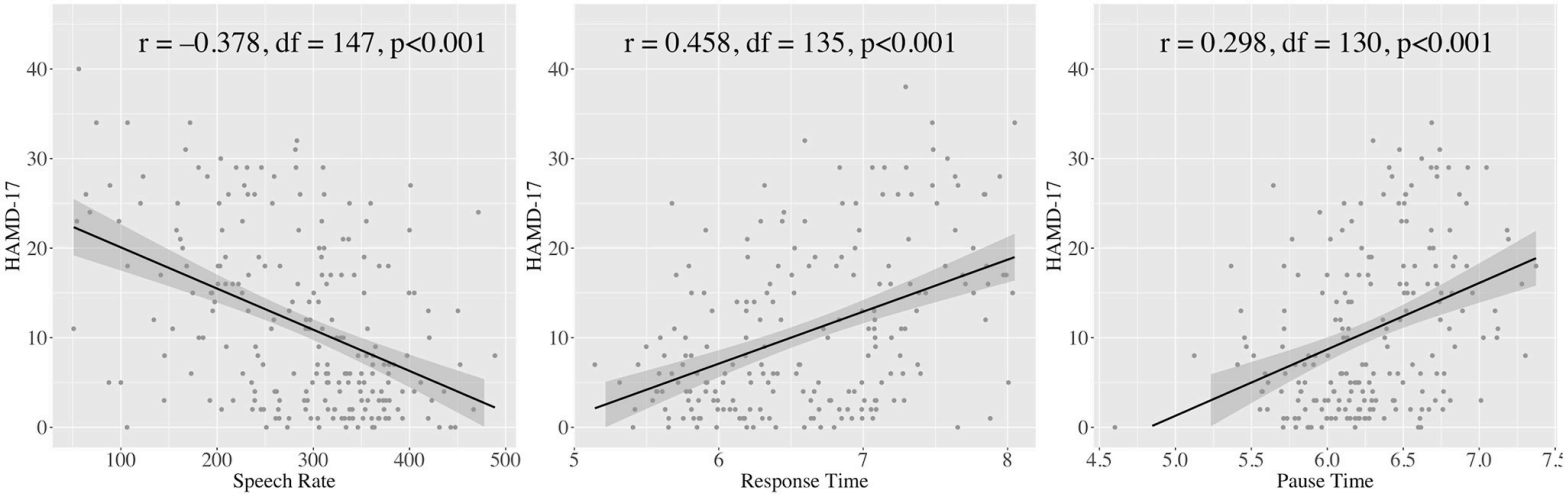
Scatter plots of speech rate, pause time, and response time.

There were significant partial correlations between HAMD-17 scores and speech rate (r = –0.378, df = 147, p <0.001), pause time (r = 0.298, df = 130, p = 0.001), and response time (r = 0.458, df = 135, p <0.001) (Fig 2).

There were still significant partial correlations between HAMD-17 scores and these timing-related speech features after excluding BP patients with YMRS higher than 8 and when patients were divided into MDD and BP respectively (S1 File).

In addition, there were significant correlations between the item 8 of HAMD-17 (psychomotor retardation) and speech rate (Spearman’s ρ = –0.242, df = 151, p = 0.003), pause time (Spearman’s ρ = 0.206, df = 135, p = 0.017), and response time (Spearman’s ρ = 0.306, df = 132, p < 0.001).

### Association between longitudinal change in HAMD-17 scores and change in timing-related speech features

There was a significant partial correlation between changes in the total HAMD-17 scores and changes in the speech rate (r = –0.317, df = 125, p <0.001). Changes in the pause time (r = 0.207, df = 107, p = 0.033) and changes in the response time (r = 0.207, df = 109, p = 0.034) were also correlated with changes in the HAMD-17 total scores.

## Discussion

To the best of our knowledge, this is the largest study to date that investigated the association between timing-related speech features and depression severity. We found that speech rate, pause time, and response time had significant correlation with the severity of depression. Moreover, longitudinal changes in these timing-related speech features showed association with longitudinal changes in total HAMD-17 scores. We acquired speech data during non-structured interviews; therefore, our results may be easier to generalize for application in daily clinical settings compared to previous studies.

### Previous studies about timing-related speech features

Speech rate has been regarded as one of the features that show promise for characterizing depression. Many initial studies reported that patients with MDD spoke slower than controls [11, 12, 17]. One study, which used audio tape recordings of seven individuals, reported that speech rate showed a significant negative correlation with HAMD scores [13]. Another study found that speech rate became faster after antidepressant treatments for patients with MDD [14]. Pause time has also been reported to be associated with depression. Previous studies in the 1970s found that pause time was longer for depressed patients than for controls, and that pause time correlated with HAMD scores [7, 11–13, 18]. Response time was analyzed in only a few previous studies [19–21]. Those studies used standard reading tasks or semi-structured interviews to gather data, and found that response time was longer for depressed patients than for controls, and that response time became shorter as HAMD scores decreased. Although these findings are in line with our findings, many previous studies have struggled to separate the voices of study participants and researchers, as well as to measure the speed and/or timing of speech. For example, some studies interviewed participants over the telephone to collect voice data [7, 14], while others had participants read fixed-form sentences in order to extract only the participant’s voice [12]. Regarding the measurement of speech timing or speed, some previous studies used an oscilloscope and a cine camera to measure the length of the utterance [11, 12], and others semi-automatically detected the occurrence of an utterance or silence using software which could not automatically detect syllables [13, 20]. Such methodological limitations made it difficult to assess large datasets. In the current study, by using array microphones and automatic speech recognition technology, we automatically obtained the timing-related speech data in real-world clinical settings from larger datasets.

### Association with psychomotor retardation

In his 1921 book, Kraepelin wrote about speech characteristics in depressed individuals: “patients speak in a low voice, slowly, hesitatingly, monotonously, sometimes stuttering, whispering, try several times before they bring out a word, become mute in the middle of a sentence. They become silent, monosyllabic, can no longer converse.” These speech characteristics are now considered to be aspects of psychomotor retardation, which is regarded as one of the prominent features of depression [22]. Assessment of psychomotor disturbances may have tremendous value for clinicians because psychomotor retardation can be a predictor for clinical response to electroconvulsive therapy and medications [23–25]. Therefore, an accurate and objective measurement of patient’s psychomotor retardation could contribute to the improvement of the classification of depression, longitudinal monitoring, and prediction of outcomes in patients with depression.

For now, there are three major scales for psychomotor retardation currently available: the Salpetriere Retardation Rating Scale (SRRS) [26], the Motor Agitation and Retardation Scale [27], and the CORE measure [28]. However, these scales are completely dependent on the subjective judgement of clinicians. In the present study, by using speech recognition technology, we were able to measure patient’s speech features in clinical settings easily and objectively. In these ways, timing-related speech features, such as speech rate, pause time, and response time, could be utilized as objective biomarkers for psychomotor retardation. As additional analyses, we investigated the relationship between HAMD-17 psychomotor retardation score and speech features and there were significant correlations between them. It would be beneficial in the future to investigate if these speech features are correlated with more detailed psychomotor retardation assessment like the CORE measure.

The process of speech production involves simultaneous cognitive planning and complex motoric muscular actions [3], so both cognitive and motor impairments can influence the process of speech production. Meanwhile, the neurobiological process underlying the inhibition of activity includes functional deficits in the prefrontal cortex and basal ganglia [22]. Structural imaging studies suggest that patients with depression have frontostriatal abnormalities, such as deep white matter lesions, and decreased volumes of the prefrontal cortex, the caudate nucleus, and the putamen [28–30]. These deficits may be more profound in the presence of psychomotor retardation [22]. The prefrontal cortex plays a fundamental role in working memory [31, 32], which is important to manipulate information and perform complex cognitive tasks for speech production. In addition, the basal ganglia form a major center in the complex extrapyramidal motor system. The striatum receives inputs from all cortical areas and, through the thalamus, projects signals principally to the frontal lobe areas, which are concerned with motor planning [33, 34]. Given this evidence, speech disturbances in patients with psychomotor retardation might be caused by functional deficits in the prefrontal cortex and the basal ganglia. As discussed above, timing-related speech features could reflect the status of neurological functions in patients with depression. This discussion is only speculative, however, future studies should investigate the relationship between speech disturbances and these neurobiological functional deficits, which may possibly lead to uncovering the pathophysiology of depression or developing better treatments.

### Limitations

Some limitations should be acknowledged. First, speech features may be influenced by the personality or speech habits of each participant. However, the results of our within-subject longitudinal analyses also suggest that speech features reflect depression severity. Second, in the current study, healthy participants were significantly older than patients with MDD or BP. However, we included age in the analyses as covariates and, despite the fact that older people generally speak more slowly than younger people [35], our results showed that speech rates were still faster in healthy controls than patients with MDD. Third, noises in the examination room may have disrupted the accuracy of the automatic speech recognition technology, although the interviews were conducted in a quiet room of the hospital. Finally, some side effects of drugs, such as sleepiness, might be related to timing-related speech features. We did not investigate detailed side effect profile in the current study to explore these complex correlations.

In conclusion, depressed individuals demonstrated a slower speech rate, longer response time, and longer pause time compared to healthy controls. Moreover, changes in these timing-related measurements were associated with depressive improvement. Our results suggest that timing-related speech features could be used as noninvasive, easy-to-use digital biomarkers for the assessment of depression severity.

## Supporting information

S1 File (DOCX)
