# Supplementary material for: Using speech recognition technology to investigate the association between timing-related speech features and depression severity
Source: PLoS One. 2020 Sep 11;15(9):e0238726. doi: 10.1371/journal.pone.0238726 (PMC7485753; doi:10.1371/journal.pone.0238726)
Supplement: S1 File — (DOCX) [file pone.0238726.s001.docx]

Supplementary Material

*Association between HAMD-17 scores and timing-related speech features in MDD and BP*

In the current study, we set YMRS scores of ≥8 and HAMD-17 scores of ≥8 as manic and depressive states, respectively. In our cohort, 14 of 68 patients with BP showed YMRS scores of >8. We conducted the correlation analyses after excluding these patients because they could confound our results. As a result, there were significant partial correlations between HAMD-17 scores and speech rate (r = –0.336, df = 132, p < 0.001), pause time (r = 0.278, df = 116, p = 0.002), and response time (r = 0.400, df = 114, p < 0.001).

We also conducted the same analyses after dividing patients into MDD and BP groups. In the MDD cohort, there were significant partial correlations between HAMD-17 scores and speech rate (r = –0.265, df = 78, p = 0.019), pause time (r = 0.277, df = 67, p = 0.022), and response time (r = 0.369, df = 65, p = 0.004). In the BP cohort, there were significant partial correlations between HAMD-17 scores and speech rate (r = –0.458, df = 63, p < 0.001), pause time (r = 0.282, df = 58, p = 0.029), and response time (r = 0.446, df = 57, p < 0.001). In BP patients presenting with YMRS scores of <8, there were significant partial correlations between HAMD-17 scores and speech rate (r = –0.460, df = 49, p = 0.001) and response time (r = 0.408, df = 44, p = 0.005), while there was no significant partial correlation between HAMD-17 scores and pause time (r = 0.217, df = 44, p = 0.147).
